# Exosomes in atherosclerosis: performers, bystanders, biomarkers, and therapeutic targets

**DOI:** 10.7150/thno.56035

**Published:** 2021-02-15

**Authors:** Chen Wang, Zhelong Li, Yunnan Liu, Lijun Yuan

**Affiliations:** Department of Ultrasound Diagnostics, Tangdu Hospital, Fourth Military Medical University, Xi'an 710038, People's Republic of China.

**Keywords:** exosomes, atherosclerosis, intercellular communication, biomarker, therapy

## Abstract

Exosomes are nanosized lipid vesicles originating from the endosomal system that carry many macromolecules from their parental cells and play important roles in intercellular communication. The functions and underlying mechanisms of exosomes in atherosclerosis have recently been intensively studied. In this review, we briefly introduce exosome biology and then focus on advances in the roles of exosomes in atherosclerosis, specifically exosomal changes associated with atherosclerosis, their cellular origins and potential functional cargos, and their detailed impacts on recipient cells. We also discuss the potential of exosomes as biomarkers and drug carriers for managing atherosclerosis.

## Introduction

Atherosclerosis involves the formation of fibrofatty lesions or plaques in the artery wall. This disease causes substantial morbidity and mortality worldwide 1, 2. The pathological process of atherosclerosis involves endothelial damage, lipid deposition, inflammatory cell infiltration, foam cell formation, and plaque formation 3, 4. Rupture of the vulnerable plaque causes *in situ* thrombosis and intramural hemorrhage, which result in ischemia and stroke 1, 5, 6.

Cellular communication is essential for nearly all physiological and pathological processes, including atherosclerosis 7. Besides their widely accepted involvement in neurotransmission and endocrine signaling, extracellular vesicles (EVs) have been recognized as new players in intercellular communication 8. EVs are classified into exosomes, apoptotic bodies, microvesicles, ectosomes, and other vesicles. Exosomes (40-160 nm in diameter 9-11) are secreted by nearly all cell types and carry biological molecules such as DNAs, RNAs, proteins, lipids, and metabolites 11. The encapsulated biomolecules not only reflect the identity of the donor cell, they also have functions in the recipient cells 9. Exosomes also display profound advantages for crossing biological barriers 12, 13, and are involved in intercellular communication over both short and long distances 14. For these reasons, exosomes have been intensively studied as biomarkers and drug carriers for diagnostic and therapeutic applications 10, 15.

Exosomes have been found to be secreted by endothelial cells, cardiac progenitor cells, cardiac fibroblasts, and cardiomyocytes, suggesting their involvement in cardiovascular diseases 9, 16, 17. For example, exosomes derived from endothelial cells have been found to play a central role in the phenotype switch of vascular smooth muscle cells (VSMCs) 18. In addition, circulating exosomes released from platelets, erythrocytes, leukocytes, and endothelial cells carry biomolecules reflecting the identity of their donor cells and so can serve as biomarkers for diverse pathological states, including atherosclerosis 19, 20. Changes in exosome levels and cargos have been reported in a variety of diseases associated with vascular injury 21-23. It has been also suggested that extracellular vesicles, including exosomes, are involved in the microcalcification in atherosclerosis 24. In general, significant changes of exosomes could be seen in atherosclerosis and associated risk factors. In turn, exosomes might function as performers, bystanders, biomarkers, and even therapeutic vehicle in atherosclerosis.

In this review, we summarize advances in the roles of exosomes in atherosclerosis. The potential of exosomes as diagnostic biomarkers and therapeutic drug carriers for atherosclerosis management are also discussed.

## Biogenesis and composition of exosomes

### Biogenesis of exosomes

Exosome biogenesis starts with invagination of the endosomal membrane, which forms a multivesicular body (MVB) inside the endosome 25. During this process, cytosolic nucleic acids and proteins are incorporated into MVBs 26. The encapsulated cargos are either degraded when MVBs fuse with lysosomes, or secreted in exosomes when endosomes fuse with the membrane of the parental cell 27-29. Since endosomes result from budding of the plasma membrane, this double-invagination process produces exosomes with the same membrane protein orientation as that of the parental cell 11, 25.

### Composition of exosomes

#### Nucleic acids

Exosomes have an aqueous core and a lipophilic shell, therefore they encapsulate hydrophilic cargos 30. Nucleic acids in exosomes have been intensively studied, mainly focusing on their roles in mediating communication between cells and their potential as diagnostic biomarkers 31. Among the exosomal RNAs, miRNAs are the most abundant type 32. Exosomal miRNAs related to atherosclerosis will be discussed in more detail later in this review. Besides the intensively studied miRNAs, a broad range of lncRNAs and circRNAs have also been identified in exosomes 33, 34. Similar to exosomal miRNAs, exosomal lncRNAs and circRNAs can also induce a series of phenotypic changes in recipient cells 35, 36.

lncRNAs are a novel group of mediators defined as long noncoding ribonucleic acids of more than 200 nucleotides. lncRNAs actively participate in biological and pathological processes 37, 38, including in cardiovascular diseases 39-41. For example, the lncRNA *NEXN-AS1* was found to regulate endothelial cell activation and monocyte adhesion via the* TLR4/NF-κB* pathway to deter atherogenesis 42. In addition, the lncRNA *CCL2* may contribute to human atherosclerosis via positively regulating *CCL2* mRNA levels in endothelial cells 43. Recent studies have also shown that lncRNAs carried by exosomes play critical roles in intercellular communication 44-47. Although the involvement of exosomal lncRNA in the regulation of cardiovascular diseases has received considerable attention, their roles in vascular dysfunction and atherosclerosis still need to be explored 39, 48.

circRNAs are covalently closed biomolecules produced by precursor mRNA back-splicing with tissue-specific and cell-specific expression patterns. circRNAs have been the highlight of recent studies 49, 50. circRNAs play regulatory roles in biological functions, such as “sponge”-like sequestration of miRNAs or proteins, and modulation of protein transcription, function, and even translation to produce polypeptides 51-53. Moreover, circRNAs have been implicated in many diseases, especially cancer and cardiovascular diseases 54, 55. The circRNA *hsa_circ_0003575* was found to be involved in oxidized low-density lipoprotein (ox-LDL)-induced endothelial cell proliferation and angiogenesis 56. Recently, involvement of exosomal circRNAs in cardiovascular functions and diseases has been increasingly reported 57. For instance, plasma exosomal *hsa_circ_0005540* was found to be a promising diagnostic biomarker of coronary artery disease 58. Further, exosomal *circHIPK3* was found to participate in the regulation of cardiac vascular injury and angiogenesis after myocardial infarction, suggesting a new mechanism of cellular communication in cardiovascular diseases mediated by exosomal circRNA 34, 59. In addition, increased *circ_0003204* in extracellular vesicles was found to stimulate ectopic endothelial inactivation in cerebrovascular atherogenesis 60. lncRNAs and circRNAs associated with cardiovascular disease are listed in **Table 1**.

Recently, exosomal mRNAs were also found, and these could be translated into proteins when exosomes are endocytosed by recipient cells 61. Notably, although dsDNA and associated histone were found in exosomes 62, this idea was challenged in a recent study, in which the authors claim that the extracellular DNA and histones were secreted independent of exosomes 63.

#### Proteins

Exosomes contain abundant proteins irrespective of their cell origin, including transmembrane proteins and cytosolic proteins 64-66. Exosomes are enriched in integrins and tetraspanins, such as CD63, CD81, CD9, and CD82 67, and cytosolic proteins, such as RAB proteins and TSG101 9, 68. In addition, many proteins participating in MVBs formation can also be found in exosomes, such as ALIX and flotillin, and these proteins are categorized as non-specific exosomal proteins 9. Additionally, heat shock proteins (HSP70 and HSP90), and cytoskeleton proteins (actin, myosin, tubulin) can also be encapsulated in exosomes 69, 70. In contrast, exosomes are free of proteins not associated with plasma membranes or endosomes, such as protein components of the endoplasmic reticulum, Golgi, mitochondria, or nucleus 71, 72. In addition, cytokines are also rarely seen in exosomes 73. Appearance of these exclusive proteins might suggest impurities in the isolated exosomes 74.

#### Lipids

The exosomal membrane lipid components are similar but slightly different from the plasma membrane of the donor cells. Ceramides, phosphatidylethanolamines, phosphatidylserines, diacylglycerides, cholesterol, sphingomyelins, and lyso-bisphospatidic acid, have been found in exosome membranes 75, 76. Notably, specific lipids are enriched in exosomes compared with donor cells and other types of EVs. For example, sphingolipids, cholesterol, and phosphatidylserines are enriched in exosomes. In addition, exosomes have a higher lipid order and thus are more resistant to detergents 77. Exosomal lipids play important roles in the biology of these vesicles, modifying the phenotype of receiving cells 78. Moreover, the lipid components might also serve as diagnostic biomarkers, with the advance of lipidomics.

## Exosome isolation methods

Current conventional exosome isolation methods include differential ultracentrifugation (UC), immunoaffinity capture and microfluidics, polymer-based precipitation, ultrafiltration (UF), and size exclusion chromatography (SEC) 25, 79. These methods have different efficiencies and purities; it is thus important to note the isolation method used when integrating data from various studies. UC can isolate exosomes from various particles, including pelleted cells, debris, and most large extracellular vesicles, by high centrifugal forces of at least 100,000 ×*g* 80. But this method cannot achieve absolute separation of exosomes, meaning that clumps of EVs, protein aggregates, and even viruses are mixed together in “isolated exosomes” samples 79. Though UC is time-consuming, labor intensive, and inefficient, it is suitable for exosomes separation of large laboratory samples 81. However, its application is limited for clinical samples 81, 82. Immunoaffinity capture and microfluidics, due to its higher capture efficiency and greater sensitivity, is an attractive approach for isolating exosomes. Its disadvantages include marker-dependent related omission and high cost 83. Precipitation methods are usually based on polyethylene glycol (PEG), a nontoxic and nondenaturating water-soluble polymer 84. This method is simple, rapid, and easy and does not require costly or specialized equipment; however, the final exosomes pellet is contaminated due to the low specificity of PEG in isolating other extracellular vesicles or proteins 85, 86. UF is an emerging size-based isolation method that uses membrane filters of defined exclusive criterion to prepare highly pure and concentrated exosomes samples with high recovery. However, it is difficult to avoid protein contamination in the exosome pellet 81, 86, 87. Accumulated evidence suggests that SEC is an ideal exosome isolation technique that can separate exosomes from most proteins to acquire pellets with low levels of contaminants and co-precipitates 88. SEC is noteworthy for its superior isolation of pure exosomes from human body fluids, and is not limited by sample volume or type, indicating its great potential to generate a high yield of exosomes for clinical and commercial applications 79. But SEC cannot distinguish exosomes from other vesicles of similar size, and it is limited by the number of samples that can be processed at one time 85, 88. Considering sample purity, cost, efficiency, and labor, UC is still the most appropriate and standard technology for exosomes isolation 81, 82. Notably, there is unneglectable overlap in particle size and density between exosomes and other non-vesicular contaminants, such as lipoproteins and nucleoproteins 89-91. Therefore, the major challenge in exosomes isolation remains the need to develop simple, cheap, and rapid methods that not only maintain the viability and features of exosomes but also distinguish them from other substances 92. Very recently, several promising methods have been developed, such as ExoTIC (exosome total isolation chip) 93, acoustofluidic platform (an integration of acoustics and microfluidics) 94, and alternating current electrokinetic microarray chip devices 95.

## Exosomal changes related to atherosclerosis risk factors

Hypertension, obesity, lipid disorder, and diabetes mellitus are major risk factors for atherosclerosis 96, 97. Accumulating studies have linked these risk factors with changes in exosome biogenesis and cargo. Cigarette smoking is also a risk factor for atherosclerosis, and future work exploring the link between smoke and exosomes are of great interest. Currently, miRNA cargos have been intensively studied, whereas exosomal lncRNAs/circRNAs are not well defined. The altered exosomal components might be important regulators of atherosclerosis, and thus the exosomal changes should be useful for predicting the risk of atherosclerosis. Moreover, therapeutic targeting these molecules might be a strategy to reduce the risk of atherosclerosis. In this section, we will focus on the relationships between atherosclerosis risk factors and exosomes.

### Exosomal changes upon hypertension

Hypertension is a primary risk factor for atherosclerosis 98. Recent studies suggest that exosomes mediate pathological processes of hypertension along with related injuries to organs 99. Circulating exosomal miRNA was found to be altered in patients with obstructive sleep apnea and hypertension, suggesting that fluctuating high blood pressure may change plasma exosome mass and cellular exchange of information 100. Exosomes have also been found to promote the development of hypertension. For example, Osada-Oka et al. showed that macrophage-derived exosomes at least partially contributed to inflammation of endothelial cells under hypertensive conditions 101. In contrast, plasma exosomes were found to modestly regulate systemic blood pressure by rebuilding the structure and function of cardiovascular tissues *in vivo* 102. Thus, elucidating the precise role of exosomes in hypertension might provide new therapeutics for hypertension and related cardiovascular diseases 103.

### Exosomal changes upon obesity

Obesity is an independent risk factor that severely threatens human life and health. With its increasing prevalence worldwide, obesity has become a serious public health challenge 104, 105. Adipose tissue not only stores lipids but also serves as an endocrine organ. Obesity is characterized by an imbalance in the adipose secretome, with an increase in proinflammatory adipocytokines and a decrease in anti-inflammatory adipocytokines 106, 107. Among the secretome, exosomes secreted by adipose tissue play key roles in whole-body glucose and lipid metabolism 108. For example, the adiponectin/T-cadherin system was found to quantitatively increase exosome biogenesis and secretion 109. Thomou et al. observed that adipose tissue significantly modulates the plasma mass of exosomes and circulating exosomal miRNAs, which regulate the expression and translation of target mRNAs in distant recipient tissues as a novel form of adipokine 110. Exosomal miRNAs have also shown robust changes in animal models of obesity. Treatment of lean mice with exosomes from obese mice, which mainly contained *miR-122*, induced metabolic dysfunction with glucose intolerance and insulin resistance 111. It is clear that adipose-derived exosomes constitute a previously undescribed class of signaling moieties, opening an avenue to better understand the pathophysiology and treatment of obesity and associated diseases 110, 112, 113.

### Exosomes in lipid disorder

Plasma lipid level is strongly associated with risk of cardiovascular disease, according to mounting prospective observational studies worldwide 3, 114-116. Blood lipid disorder is an accepted causal risk factor for atherosclerosis, especially in plaque progression and thrombosis 96, 117. Recently, many studies have focused on the relationship between exosomes and lipid disorder. Exosomes-mediated lipid metabolism covers the process of lipid synthesis, transportation, and degradation, which have been implicated in atherosclerosis 118. For example, exosomes are an adequately potent source of eicosanoids such as prostaglandins and leukotrienes, both of which are active *in vivo* and *in vitro*. The biological significance and mechanism of exosomal shuttling in the eicosanoids synthesis pathway has attracted rapidly growing interest 119. In addition to transporting lipids directly to recipient cells, exosomes can also regulate the expression of classical lipid transporters, such as reverse cholesterol transport mediated by ABCA1 120. Furthermore, substantial evidence suggests that brown adipose tissue (BAT)-derived exosomes can alleviate lipid accumulation and improve cardiac function, indicating that exosomes are involved in lipid degradation and adipose tissue redistribution 121. In turn, growing evidence suggests that lipid metabolism affects the biological functions of exosomes, including bioprocesses from signal transduction by receptor-ligand interactions and exosome internalization by or fusion with recipient cells, which provides a new perspective for better understanding the occurrence and development of atherosclerosis 118.

### Diabetes mellitus associated exosomal changes

Numerous studies have shown that diabetes is associated with accelerated atherosclerosis and that exosomes have pathophysiological effects on atherosclerotic plaque destabilization 122, 123. Patients with type 1 diabetes mellitus (T1DM) have increased plasma levels of exosomes. Upregulation or downregulation of exosomal miRNAs is associated with progression of this disease 124, 125. Karolina et al. revealed that four exosomal miRNAs (*miR-17*, *miR-197*, *miR-509-5p*, and *miR-92a*) were reduced while *miR-320a* was increased in patients with type 2 diabetes mellitus (T2DM) 126. Moreover, these altered exosomes might in turn promote the development of atherosclerosis. Wang et al. determined that insulin-resistant adipocyte-derived exosomes accelerated atherosclerosis and plaque vulnerability by inducing vasa vasorum angiogenesis 127. Moreover, insulin resistance has been reported to drive extracellular vesicles secretion, which may contribute to the quantitative alteration of plasma exosomes in diabetes, and highlights their potential as diagnostic tools of T2DM 128.

## Exosomes in atherogenesis

Accumulating evidence has revealed that exosome-mediated cellular interactions play important roles in atherogenesis 19, 129. The effects of exosomes on atherosclerosis are intensively discussed in a recent excellent review 130. In this section, we will focus on the various origins of exosomes in atherosclerosis and the underlying mechanisms involved.

### Biological functions of exosomes

**Figure 1:**
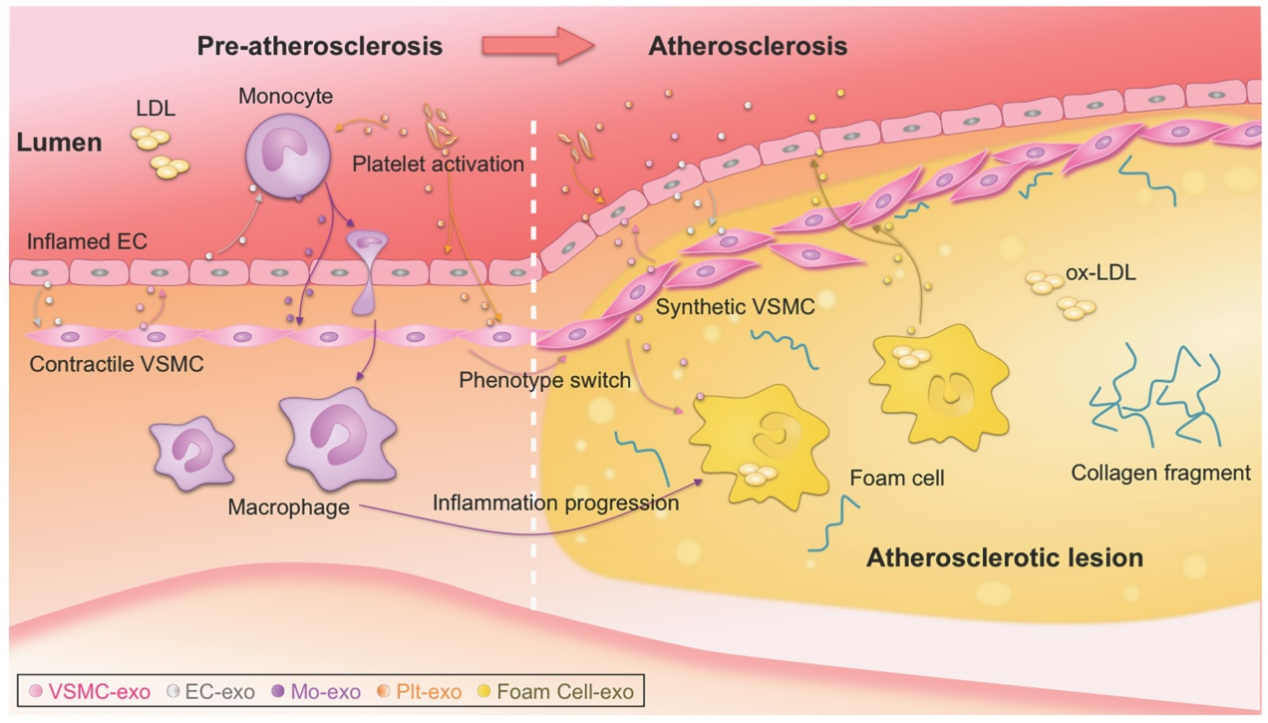
** Exosome-mediated intercellular communication in the progression of atherosclerosis.** Exosomes could be secreted by all types of the cells involved in atherosclerosis, such as monocytes, macrophages, platelet, endothelial cells (ECs), vascular smooth muscle cells (VSMCs). Intercellular communication via exosomes occurs, transmitting signal from one cell type to another, contributing to the progression of atherosclerosis. Detailed roles of the exosomes from different origins should be different, and thus the exosomes act as either bystanders or performers in the process. ox-LDL, oxidized low-density lipoprotein.

Atherosclerotic lesions are initiated by the accumulation of low-density lipoprotein (LDL) particles in the intima, adhesion of blood monocytes to the injured endothelium, migration of the monocytes into the intima, and maturation of macrophages along with the formation of lipid-filled foam cells 3. Phenotype switching of VSMCs from contractile to synthetic type and chronic inflammation of the arterial wall also drive the progression of atherosclerosis. With the progression of atherosclerosis, a necrotic core and thrombosis ultimately form in the lesion 1, 2. Recently, the notion that plaque healing may play a key role in the natural history of atherosclerotic disease has updated traditional theories of atherosclerosis 117. Notably, exosomes have been reported to be actively involved in nearly all the above biological processes 19, which is a new, dynamic area of research (**Figure 1**) 131, 132. It is also important to note that there is far less than one molecule of a given RNA per exosome, even for the most abundant miRNAs. This stoichiometry of miRNAs and exosomes suggests that most individual native exosomes either from pathological or physiological conditions do not carry biologically significant numbers of RNAs. Thus, individual exosome is unlikely to be functional as vehicle to transfer functional RNAs 133. In other words, the observed pathophysiological effects might stem from that amounts of exosomes of similar function work together for a long duration.

### Origins of exosomes

#### Endothelial cell-derived exosomes

Endothelial dysfunction is the initial step in the process of atherogenesis 134-136. Endothelium has important functions in the regulation of inflammation, coagulation, vascular tone, and vascular wall permeability. Endothelial dysfunction triggers release of extracellular vesicles, including exosomes 137. Moreover, cellular stress conditions are reflected in exosomal protein and RNA 22. Endothelial cell-derived exosomes are involved in atherogenesis by transferring biological messages to other cells 138. Endothelial cell-derived vesicles regulate VSMC phenotype via their cargos 139. For example, *miR-143/145*-containing extracellular vesicles derived from KLF2-expressing endothelial cells reduced atherosclerotic lesions in *ApoE^-/-^* mice 140. Similarly, endothelial cell-derived exosomes could inhibit the VSMC phenotype switch 141. Moreover, exosomes of endothelial origin can modulate monocyte activation by transferring *miR10a* 142. The involved exosomal miRNAs are summarized in **Table 2**. Furthermore, some exosomal lncRNAs and circRNAs have also been found in endothelial cell-derived exosomes. Exosomes from ox-LDL-treated endothelial cells induced dendritic cell maturation in atherosclerosis due to loss of the lncRNA *MALAT1* (**Table 1**) 143.

Recent evidence suggests that exosomes derived from endothelial progenitor cells (EPCs) may participate in the repair of endothelial function at some stage 144. As the precursor cells of vascular endothelial cells, EPCs are a type of stem cell from the bone marrow with limited differentiation ability and strong growth ability 145. Exosomes derived from EPCs regulate VSMC phenotype via the *ACE2/NF-κB/Ang II* pathway, indicating their potential for hypertension treatment 146. Meanwhile, it has been revealed that EPC-derived exosomes overexpressing angiotensin-converting enzyme 2 (ACE2) can protect endothelial cells by decreasing apoptosis and improving mitochondrial function 147. Furthermore, EPC-derived exosomes significantly decreased the production of atherosclerotic plaques and inflammatory factors, and ameliorated endothelial dysfunction in a mouse model of atherosclerotic diabetes 148. Conversely, a study showed that EPC-derived exosomes have attenuated myocardium repair properties due to enrichment of exosomal integrin-linked kinase under IL-10 deficiency or inflammation conditions, which indicates the potential of exosomal protein manipulation as an advanced therapeutic method for cardiovascular diseases 149.

#### VSMC-derived exosomes

VSMCs below the endothelium control vascular tension at physiological conditions 2. VSMC-derived exosomes are novel critical regulators of vascular hemostasis 150, 151. *miR-1246*, *miR-182*, and *miR-486* in VSMC-derived exosomes play essential roles in the maintenance of vascular homeostasis 152. Numerous studies have shown that proliferation, phenotype switching (mainly contractile to migratory state), apoptosis, and calcification of VSMCs are closely linked to the onset and progression of atherosclerosis 153-155. In the process of atherosclerosis, VSMCs communicate with surrounding cells by secreting various factors, with exosomes emerging as a new mediator (**Figure 1**) 156. Under pathological conditions, VSMCs switch to the synthetic phenotype and actively secrete exosomes to induce endothelial migration and angiogenesis, promoting the formation of atherosclerotic plaques and triggering vascular calcification 155, 157. In addition, exosomes from calcifying VSMCs were found to accelerate calcification by propagating procalcifying signals. Moreover, proliferating VSMCs were found to release more exosomes and exosomes were found deposited in precalcified vessels, which may prime the vessel wall to calcify 150, 158, 159. Theoretically, preventing release of exosomes from calcified VSMCs might effectively prevent vascular calcification and the formation of atherosclerotic plaques (**Figure 1**) 157, 160.

miRNAs are considered to be the main functional cargos of VSMC-derived exosomes. For example, exosomes derived from KLF5-overexpressing VSMCs were found to transfer *miR-155* to endothelial cells, which in turn inhibited endothelial cell proliferation and migration, eventually impairing tight junctions and the integrity of endothelial barriers 161. VSMC-derived exosomal miRNAs involved in atherogenesis are summarized in **Table 2**. Besides miRNAs, circRNAs are also involved. For example, *hsa_circ_0001445* was found to be downregulated in extracellular vesicles secreted by coronary smooth muscle cells in atherogenic conditions, which could be used as a biomarker to improve the identification of coronary artery atherosclerosis 162.

#### Inflammatory cell-derived exosomes

Macrophages in the subendothelial space of the artery wall, which are differentiated from monocytes, are involved in all stages of atherosclerosis, from endothelial dysfunction, to lesion expansion, and formation of the plaque 163. Notably, the idea that macrophages have a diminished capacity to egress remains challenged 164, 165. Exosome biogenesis is different in macrophages and the derived exosomes could play crucial roles throughout the whole process of atherosclerosis (**Figure 1**). Macrophage-derived foam cells release more exosomes than normal macrophages 166. Inflamed macrophages secrete exosomes that promote cytokine production when endocytosed by recipient cells, which recruits other immune cells to inflamed sites 167. Exosomes derived from ox-LDL‑stimulated macrophages were found to impair endothelial function 168. Extracellular vesicles containing *miR-146a* secreted from macrophages in a proatherogenic environment functionally altered recipient cell function *in vitro*, suggesting a potential role in atherogenesis 169. Consistently, exosomal *miR-146* from atherogenic macrophages was found to deteriorate atherosclerosis development by promoting neutrophil extracellular traps 170. Besides *miR-146*, other miRNAs might also be involved. For example, exosomes from nicotine-stimulated macrophages were found to at least partially contribute to nicotine-promoted atherosclerosis, in which exosomal *miR-21-3p* promoted VSMC migration and proliferation 171. In addition, exosomal *miR-99a/146b/378a* derived from alternatively activated macrophages downregulated *TNF-α/NF-κB* signaling and alleviated inflammation 172. Besides the monocyte/macrophage derived exosomes, exosomes from neutrophil and other inflammatory cells might be also involved. The detailed functions and targets of these miRNAs are summarized in **Table 2**.

#### Platelet-derived exosomes

Platelets have emerged as potent regulators of atherosclerosis by facilitating recruitment of inflammatory cells 173-175. Heightened platelet adhesion, activation, and aggregation are pivotal pathophysiological conditions associated with the initiation and progression of atherosclerotic lesions 176, 177. Moreover, exosomes are major mediators in the crosstalk between platelets and other cells in the pathogenesis of atherosclerosis (**Figure 1**) 178. Platelet-derived exosomes are the most abundant type in the bloodstream in normal conditions 179. Activated platelet-derived exosomes were found to promote the proliferation and migration of HUVECs, shedding new light on the effects of platelet-derived exosomes in atherosclerosis and intraplaque angiogenesis 180. In contrast, platelet exosomal *miR-25-3p* was shown to inhibit ox-LDL-induced coronary vascular endothelium inflammation 181. Platelet-derived exosomes can also be uptaken by endothelial cells, where the exosomes inhibit ICAM-1 expression at least partially via *miR-223* 182, 183.

#### Circulating exosomes of other origins

Exosomes originating from a variety of cell types are released into the blood as circulating exosomes for long distance transport of biomolecules 179. Exosomes associated with atherosclerosis mainly originate from platelets, leukocytes, VSMCs, and endothelial cells, and the exosomes discussed above constitute the majority of circulating exosomes 19, 62. However, circulating exosomes can also be released from other sources. For example, adipose tissue constitutes a major source of circulating exosomes that serve as a novel form of adipokine for cellular communication and regulation 110. As a result of mutual interactions between distribution and function, alteration of adipose tissue greatly affects circulating exosomes and their cargos 110, 184. Additionally, adipose tissue is a well-established driver in the development of obesity, which is one of the most critical risk factors for atherosclerosis 185. Thus, exosomes derived from adipose tissue should actively influence atherogenesis, but the specific mechanisms remain unclear. In addition, skeletal muscle with secretory activities has been suggested to be another irreplaceable source of circulating exosomes 186, 187. The healthy state of muscle is inextricably linked to regular physical activity, which helps reduce the risk of sedentary lifestyle-induced chronic cardiovascular diseases such as atherosclerosis 188. Collectively, muscle-derived circulating exosomes play crucial roles in atherosclerosis 189. Studies profiling changes in circulating exosomes associated with atherosclerosis-related metabolic abnormalities, as well as identifying their mechanisms, would be highly valuable.

## Exosomes in atherosclerosis diagnosis and therapy

### Exosomal miRNAs as putative biomarkers

**Figure 2:**
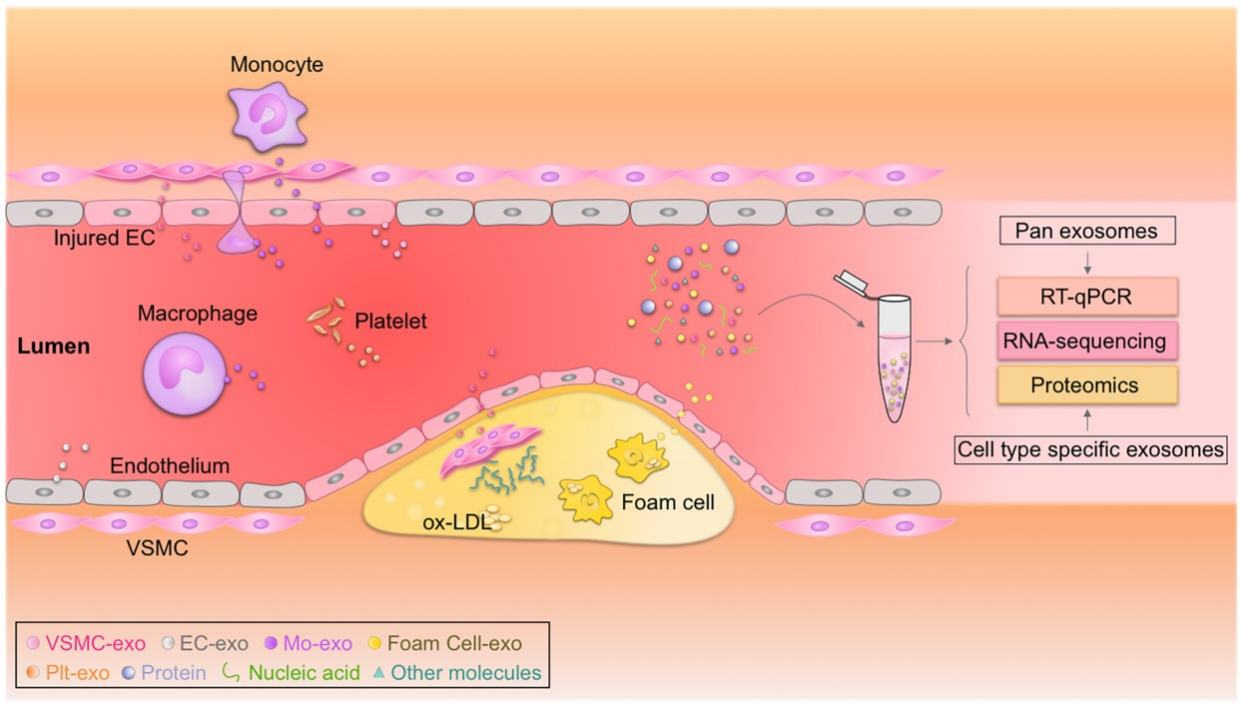
** Circulating exosomes as emerging biomarkers for atherosclerosis.** Circulating exosomes (exo) from different cell types associated with atherosclerosis carry cargos with identities similar to their donor cells. Real-time quantitative polymerase chain reaction (RT-qPCR), proteomic, and transcriptomic profiling of these biomarkers could be used to diagnose atherosclerosis. EC, endothelial cell; Mo, monocyte; ox-LDL, oxidized low-density lipoprotein; Plt, platelet; VSMC, vascular smooth muscle cell.

The discovery, validation, and implementation of novel biomarkers are important for improving prognosis in the clinic 190, 191. Exosomes have emerged as rational biomarkers for various diseases as they are easily accessible, carry disease-specific cargos, and have a high degree of stability in body fluids. Exosome-derived miRNAs can be isolated from multiple fluids faultlessly, raising exciting opportunities for clinical translation (**Figure 2**) 190, 192. Theoretically, exosome-derived miRNAs are a better biomarker than circulating miRNAs in plasma/serum, as exosomes from specific cell types can be purified, ensuring sensitivity and specificity 193, 194. Jiang et al. found that a specific circulating exosomal miRNA signature (*miR-122-5p*, *miR-27b-3p*, *miR-101-3p*, etc.) is a novel biomarker predicting recurrent ischemic events in intracranial atherosclerotic disease 195. Additionally, release of exosomal *miR-92a-3p* from endothelial cells is associated with atherogenic conditions and could serve as a potential diagnostic biomarker 196. Furthermore, plasma exosomal *miR-30e* and *miR-92a* expressions were up-regulated in atherosclerosis and negatively correlated with plasma cholesterol and ABCA1 levels, providing a new biomarker for the clinical diagnosis and treatment of coronary atherosclerosis 197. In addition, exosomal miRNAs involved in atherosclerotic lesion development, such as *miR-133a*, *miR-155*, *miR-21*, *miR-210*, *miR-126*, and *miR-499*, have also emerged as promising biomarkers for diagnosis, risk stratification, and prognosis prediction 194, 198, 199. According to a recent study by Sorrentino et al., circulating exosomes and their encapsulated miRNAs correlated well with atherosclerosis severity, suggesting a potent diagnostic potential 200. Despite these promising results, none of these biomarkers have been validated in large cohort studies. Like all other biomarkers, before exosomal biomarkers can be translated to the clinic, they must be validated and accredited by the International Organization for Standardization 201. In addition, exosome isolation methods should also be standardized 202.

### Therapeutic potential of exosomes in atherosclerosis

**Figure 3:**
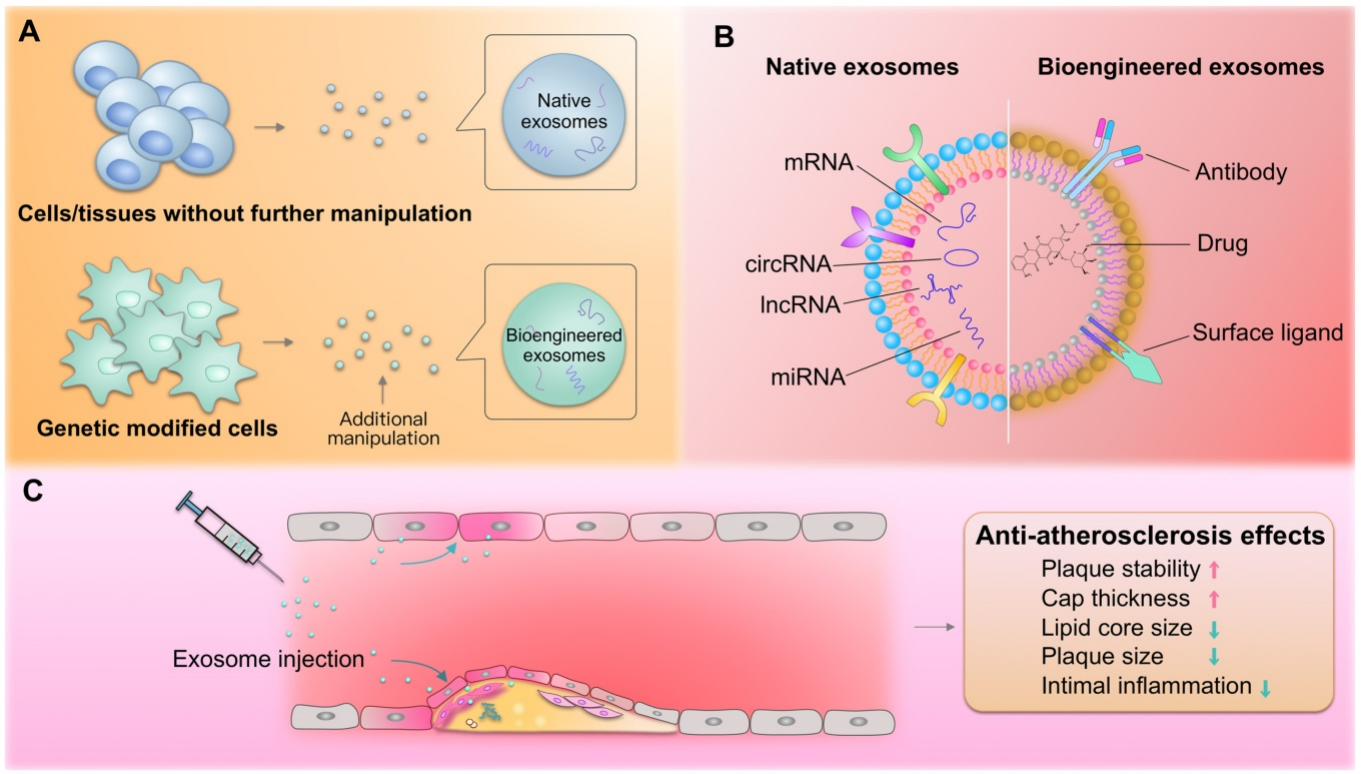
** Exosomes in atherosclerosis therapy.** (A) Both native and bioengineered exosomes are promising strategies for atherosclerosis therapy. The native exosomes are derived from cells/tissues without additional manipulation. The bioengineered exosomes could be either from gene modified cells or further modified with chemical or physical manipulation after isolation. (B) The native or bioengineered exosomes are of typical structure of EVs. Compared with other nanoparticles, the exosomes have advantages of high biocompatibility. The surface of exosomes could be engineered to harbor targeting moieties, such as antibodies or other ligands, while the inside could be engineered to encapsulate cargos of interest. (C) The delivered exosomes target various cells (endothelial cells, macrophages, etc.) involved in atherosclerosis, alleviating the pathological process.

Over the last few years, exosomes have been considered as potential biotherapeutics and drug delivery vectors for various diseases. Their natural functional nucleic acid and protein cargos have raised the possibility that exosomes from specific origins may be therapeutic drugs. For example, exosomes from cardiac stem cells could regulate cellular processes in recipient cardiac cells toward better regeneration 203. In addition, exosomes could be harnessed for the therapeutic delivery of RNAs, peptides, and synthetic drugs 204. For example, we recently established an exosome-mediated *Ldlr* mRNA delivery strategy, which could effectively rebuild *Ldlr* expression and stabilize atherosclerotic plaques in *Ldlr^-/-^* mouse model, providing a promising therapeutic approach for atherosclerosis 205. Numerous studies have explored the roles of exosomes in managing atherosclerosis. Compared with the commonly used nanoparticles, exosomes are of great advantage in low immunogenicity and evasion from the phagocytosis by macrophages. Exosomes derived from the native tissues/cells and gene modified cells, namely native and bioengineered exosomes, are promising for atherosclerosis therapy (**Figure 3A**). In addition, exosomes could be also engineered after the exosomes are isolated through click-chemistry. Generally, the exosomes could be engineered to encapsulate types of cargos with therapeutic efficacy and surface functionalized with peptides or antibodies targeting cells/tissues of interest (**Figure 3B**). The delivered exosomes target various cells (endothelial cells, macrophages, etc.) involved in atherosclerosis, alleviating the pathological process (**Figure 3C**).

Xing et al. demonstrated that exosomal *miR-342-5p* from adipose-derived mesenchymal stem cells protects endothelial cells against atherosclerosis 206. Stem cell-derived exosomes have been successfully used in animal models with demonstrated efficacy and potential benefits 207. However, the potential of stem cell-derived exosomes as drug candidates is limited by the lack of high-yield and scalable manufacturing processes for both stem cell culture and isolation 74.

Besides native exosomes, exosomes are easily manipulated to encapsulate therapeutics. For example, M2 macrophage-derived exosomes displayed effective treatment of atherosclerosis, especially when loaded with hexyl 5-aminolevulinate hydrochloride 208. However, translation of exosomes as drug delivery vehicles has been impeded by their low loading efficiencies 209, 210. In one approach to overcoming this limitation, large RNA cargos were encapsulated into exosomes by fusing the exosomal membrane protein CD9 and an RNA-binding protein together with the RNA of interest 211. In addition, systemically delivered exosomes are prone to trapping in nonspecific organs, especially the liver, lung and spleen, leading to an insufficient dose in the target area 212. Therefore, surface modifications for targeted delivery may provide opportunities to enhance or broaden the innate therapeutic capabilities of exosomes 191, 213. And a sensitive method to label exosomes with the fusion protein makes it easier to analyze the change of exosome-mass by tracking them *in vivo* 214. Surface ligand enrichment on engineered exosomes may enable the development of receptor-mediated tissue targeting, promote signaling events in recipient cells, or target exosomes to specific cell types 9, 204. Emerging bio-nanotechnologies offer promising advances in diagnostics and therapy 215. For example, hybrid nanosystems based on genetically engineered exosomes and thermosensitive liposomes are a novel strategy to improve delivery efficacy 216. Additionally, drug loading and delivery efficiency can be improved through the design of exosome-like nanovesicles and membrane-camouflaged nanoparticles 217. For example, to combine their biophysical and biomolecular advantages, gold nanoshells (which are non-cytotoxic 218) were assembled and grown on vesicles *in situ* to achieve rapid and multiplexed analysis of exosomal targets, offering a novel avenue for accurate patient prognosis and therapy 219 (**Figure 3**). In summary, we anticipate that native and bioengineered exosomes will be translated to atherosclerosis management, and we expect that exosome-like nanoparticles will become effective strategies to address current problems.

## Conclusion and outlook

Atherosclerosis and associated cardiovascular diseases are a worldwide health burden. Accumulating evidence has suggested that exosomes are important players in these diseases. Exosomes altered in the context of disease risk factors can be released and taken up by most of the known cell types in atherosclerosis 220. These exosomes not only reflect the progress of atherosclerosis but also contribute to its development, opening avenues for diagnosis and therapy.

The methods used for exosome isolation critically impact subsequent analyses. Strategies to isolate cell-specific exosomes and methods to analyze exosomal contents with high sensitivity are needed, which in turn would broaden our understanding in the field. Currently, multiple methods with varied purity are used in different labs. It is thus strongly recommended to standardize the isolation procedure before integrating studies across different labs.

There is far less than one molecule of a given RNA molecule per exosome, even for the most abundant miRNAs. Thus, the observed pathophysiological effects can only be achieved by that amounts of exosomes of similar function work together for a long duration 133. It is thus important to load amounts of cargos for therapeutic purposes. In addition, repeat intervention should be also essential for expected effects.

The various roles of exosomes from different cell types and the detailed exosomal cargos involved in atherosclerosis remain largely unknown. Beyond the commonly studied miRNAs, lncRNAs, circRNAs, and some other bioactive molecules could also be involved in the function of exosomes. For example, very long-chain acyl-CoA dehydrogenase (ACADVL), an enzyme located in mitochondria, was found to be highly enriched in exosomes derived from BAT. BAT-derived exosomes could transfer ACADVL as a functional protein into liver cells 121. Thus, the roles of exosomal proteins and lipids in atherosclerosis are emerging research areas. Procedures to implement omics approaches to conventional biological studies should also be standardized. Before clinical translation, we urgently need to confirm which exosomal components have profound diagnostic and therapeutic value, particularly as accurate biomarkers reflecting disease, membrane moieties for targeting, and key cargos involved in disease processes. We anticipate that current and future findings from profiling and mechanism studies of exosomes in atherosclerosis could be harnessed for diagnosis and therapy.

## Figures and Tables

**Table 1:** Exosomal lncRNAs and circRNAs involved in atherosclerosis

lncRNA/circRNA	Expression/Function	Target	Implication	Isolation method	Ref.
lncRNA* MALAT1*	Inhibits maturation of DCs	*NRF2*	Regulates progression of atherosclerosis	miRCURY Exosome Kit	143
*circHIPK3*	Regulates dysfunction of CMVECs	*miR-29a/IGF-1*	Shuttles with exosomes and is a potential treatment target	Ultracentrifugation	34
	Accelerates cell cycle progression and proliferation	*miR-29a/VEGF-A*	Cardioprotective	Ultracentrifugation	59
*circ_0003204*	Mediates endothelial phenotype	*miR-370-3p/TGFβR2/phosph-SMAD3*	Novel stimulator and potential biomarker	ExoQuick	60
*circ_0005540*	Elevated in patients with CAD	NA	Promising diagnostic biomarker for CAD	exoRNeasy kit	58
*circ_0001445*	Downregulated in atherogenic conditions	NA	Improves the identification of coronary artery atherosclerosis	NA	162

CAD: coronary artery disease; CMVEC: cardiac microvascular endothelial cell; DC: dendritic cell; IGF-1: insulin-like growth factor-1; MALAT1: metastasis-associated lung adenocarcinoma transcript 1; NA: not available; NRF2: nuclear factor erythroid 2-related factor; SMAD3: small mothers against decapentaplegic 3; TGFβR2: transforming growth factor β receptor 2; VEGF-A: vascular endothelial growth factor-A.

**Table 2:** Exosomal miRNAs involved in atherosclerosis

Origin	Cargo	Function	Target	Implication	Isolation method	Ref.
	*miR-10a*	Modulates monocyte activation	*NF-κB*	Represses inflammatory signal in cardiovascular disease	Medium: ultracentrifugation; Plasma: ExoQuick	142
Endothelial cell	*miR-143/145*	Controls VSMC phenotypes	*KLF2*	Reduces atherosclerotic lesion formation	Multi-step centrifugation	140
	CD137	Decreases anti-inflammatory effects	*TET2*	Accelerates VSMC proliferation and migration and neointimal formation	Exo-spin	141
	*miR-155*	Suppresses the expression of TJ proteins	*ZO-1*	Impairs endothelial barrier function	Differential centrifugation	161
	noncrystalline Ca/P salt	Promotes calcification	*SMPD3*	Calcifies vascular cells and enriches in calcified vasculature	Differential ultracentrifugation	150
VSMC	*miR-1246*	Inhibits EC migratory activities	NA	Maintains vascular homeostasis	ExoQuick-TC	152
	*miR-182*					
	*miR-486*					
Monocyte/macrophage	*miR-146a*	Reduces macrophage migration	*IGF2BP1/HuR*	Accelerates development of atherosclerosis	ExoQuick-TC, differential ultracentrifugation	169
		Promotes ROS and NETs release	*SOD2*	Slows atherosclerosis development	Medium: ExoQuick-TC; Serum: differential ultracentrifugation	170
	integrins	Changes phosphorylation levels	*ERK/AKT*	Promotes VSMC migration and adhesion	Ultracentrifugation	166
	*miR-21-3p*	Promotes VSMC migration and proliferation	*PTEN*	Accelerates atherosclerotic plaque progression	Ultracentrifugation, sucrose density gradient centrifugation	171
	*miR-99a*	Fosters M2 polarization, reduces hematopoiesis, and suppresses inflammation	*NF-κB/TNF-α*	Reduces necrotic lesion area, stabilizes atheroma, and controls atherosclerosis	Cushioned-density gradient ultracentrifugation	172
	*miR-146b*					
	*miR-378a*					
Platelet	HMGB1	Initiates a cascade of platelet thrombogenesis	NA	Biomarker of platelet abnormalities	ExoQuick-TC	178
	*miR-223*	Inhibits ICAM-1 expression	*NF-κB/MAPK*	Regulates thrombosis-inflammation reaction	Ultracentrifugation	182, 183
	*miR-126*	Promotes the proliferation and migration of HUVECs	NA	Contributes to intraplaque angiogenesis	ExoQuick-TC	180
	*miR-25-3p*	Inhibits ox-LDL-induced EC inflammation and lipid deposition	*NF-κB/Adam10*	Alleviates atherosclerosis and is a potential treatment target	ExoQuick-TC	181

Adam10, a disintegrin and metalloprotease 10; AKT, protein kinase B; ERK, extracellular regulated protein kinases; HMGB1, high-mobility group box 1 protein; HuR, human antigen R; ICAM-1, intercellular adhesion molecule-1; IGF2BP1, insulin-like growth factor 2 mRNA-binding protein 1; KLF2, krüppel-like factor 2; M2, M2 macrophages; MAPK, mitogen-activated protein kinase; NA, not available; NETs, neutrophil extracellular traps; NF-κB, nuclear factor-κB; PTEN, phosphatase and tension homologue; ROS, reactive oxygen species; SMPD3, sphingomyelin phosphodiesterase 3; SOD2, superoxide dismutase 2; TET2, ten-eleven translocation 2; TJ, tight junctions; TNF-α, tumor necrosis factor α; VSMC, vascular smooth muscle cell; ZO-1, zonula occludens-1.
